# Revealing the Electric and Magnetic Nature of the Scattered Light

**DOI:** 10.1021/acsphotonics.4c00837

**Published:** 2024-08-15

**Authors:** Jorge Olmos-Trigo

**Affiliations:** Departamento de Física, Universidad de La Laguna, Apdo. 456., E-38200 San Cristóbal de La Laguna, Santa Cruz de Tenerife, Spain; Centro de Fisica de Materiales, Paseo Manuel de Lardizabal 5, 20018 Donostia-San Sebastian, Spain

**Keywords:** nanophotonics, electromagnetic radiation, polarimetry, nanoparticles, Stokes vector method

## Abstract

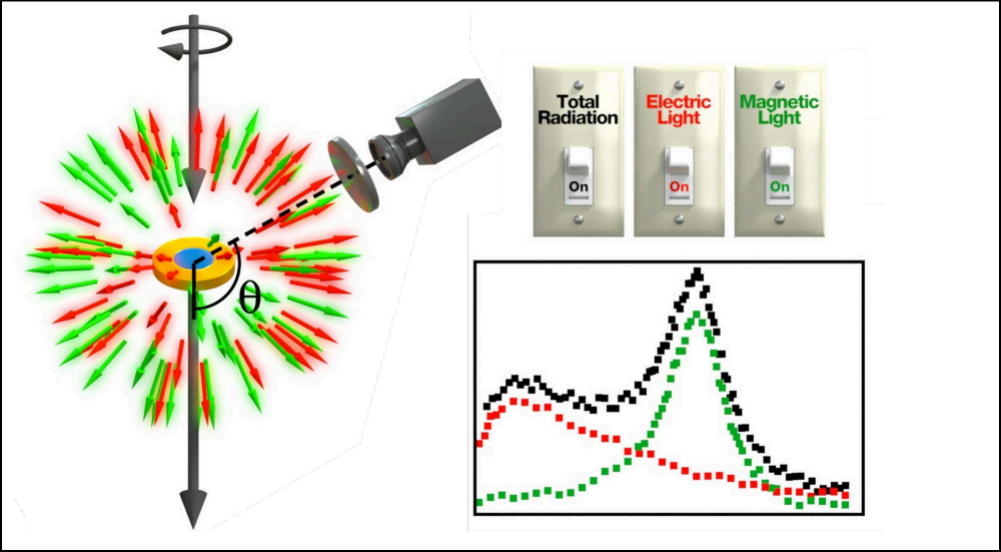

The multipolar expansion of the electromagnetic field plays a key role in the study of light–matter interactions. All the information about the radiation and coupling between the incident wavefield and the object is embodied in the electric and magnetic scattering coefficients  of the expansion. However, the experimental determination of  requires measuring the components of the scattered field in all directions, something that is exceptionally challenging. Here, we demonstrate that a single measurement of the Stokes vector unlocks access to the quadrivector . Thus, our Stokes polarimetry method allows us to capture  and  separately, a distinction that can not be achieved by measuring the total energy of the scattered field via an integrating sphere. Moreover, the determination of  enables us to infer the amplitude of the scattered field at all points of the radiation zone, including the amplitude of the near-field distribution generated by the objects. Importantly, we demonstrate the robustness of our Stokes polarimetry method, showing its fidelity with just two measurements of the Stokes vector at different scattering angles.

## Introduction

The multipolar expansion of the electromagnetic field is a key tool in the study of light–matter interactions and has historically played a pivotal role in several branches of Nanophotonics.^1^ These include optical forces,^2^ optical torques,^3^ and chiral light–matter interactions,^4^ among others.^5,6^

The multipolar expansion of the electromagnetic field is typically written as an infinite sum of electric and magnetic multipoles that are, in turn, weighted by its corresponding electric and magnetic scattering coefficients, respectively.^7^ Researchers have access to the multipolar expansion of the incident wavefield since its coefficients are known. However, the situation changes when the incident wavefield interacts with an object. In this case, the electric and magnetic scattering coefficients, denoted as  and , respectively, are unknown complex quantities and their determination is crucial to solving the scattering problem under investigation. In this setting,  and *m* denote the multipolar order and total angular momentum, respectively.^7^

From the theoretical perspective, the following multistep procedure is typically employed to retrieve : First, numerical methods are employed to obtain the components of the scattered field in all directions. Some examples of these numerical approaches are the T-matrix method,^8^ the Discrete Dipole Approximation (DDA),^9^ along with all kinds of Maxwell solvers. Subsequently, by projecting the scattered field onto the corresponding electric (or magnetic) multipole, the desired electric (or magnetic) scattering coefficient can be calculated.^7^ However, a fundamental problem arises in the previous approach to determine : it lacks experimental equivalence, primarily due to the formidable task of measuring the components of the scattered field in all directions.

**Figure 1:**
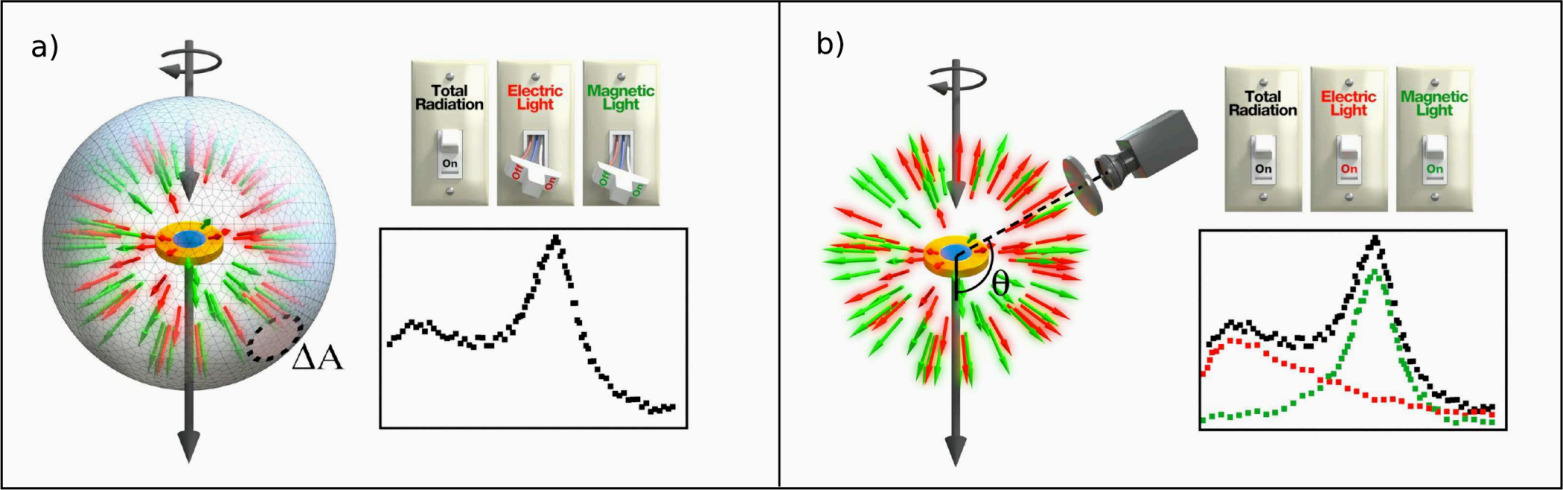
Artistic representation of the measurement of the scattering cross-section of a nanodisk under the illumination of a circularly polarized wavefield. The red and green arrows represent the electric and magnetic amplitudes of the scattering coefficients in the scattered field. (a) An integrating sphere is placed in the far-field to collect the components of the scattered field in all directions. This measurement does not allow distinguishing between the electric and magnetic amplitudes of the scattering coefficients. (b) The Stokes vector measurement, in which a photodiode and conventional waveplates are placed at a scattering angle θ, allows distinguishing between the electric and magnetic contributions to the scattering cross-section.

In this work, we present an experimentally feasible Stokes polarimetry approach that solves this experimental challenge for objects well-described by a single multipolar order  and total angular momentum *m*. More specifically, we demonstrate that a measurement of the Stokes vector grants access to all the components of the quadrivector 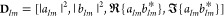
enabling the separate detection of  and . Remarkably, this distinction between the electric and magnetic amplitudes of the scattering coefficients is unreachable if measuring the scattering cross-section. To visualize this distinction, check Figure 1, where we show the scattering cross-section of a nanodisk excited by a circularly polarized wavefield. Two experimental setups are depicted to measure the scattering cross-section: an integrating sphere embedding the excited nanodisk (see Figure 1a) and our Stokes polarimetry approach in which only a photodiode and conventional wave-plates are needed (see Figure 1b). As Figure 1b shows, the Stokes polarimetry approach allows telling between  and . Importantly, in our work, we do not impose any restrictions on the spatial distribution of the incident wavefield. Accordingly, our Stokes polarimetry approach can accommodate a wide range of illumination conditions.

On top of that, by measuring the Stokes vector at two different scattering angles, we establish the fidelity of our Stokes polarimetry approach. That is, we demonstrate that our method is robust and can be trusted without performing any numerical simulation. Our findings, supported by analytical theory and exact numerical simulations, are promising for all branches of Nanophotonics, as they facilitate the characterization of objects in optical laboratories.

**Figure 2:**
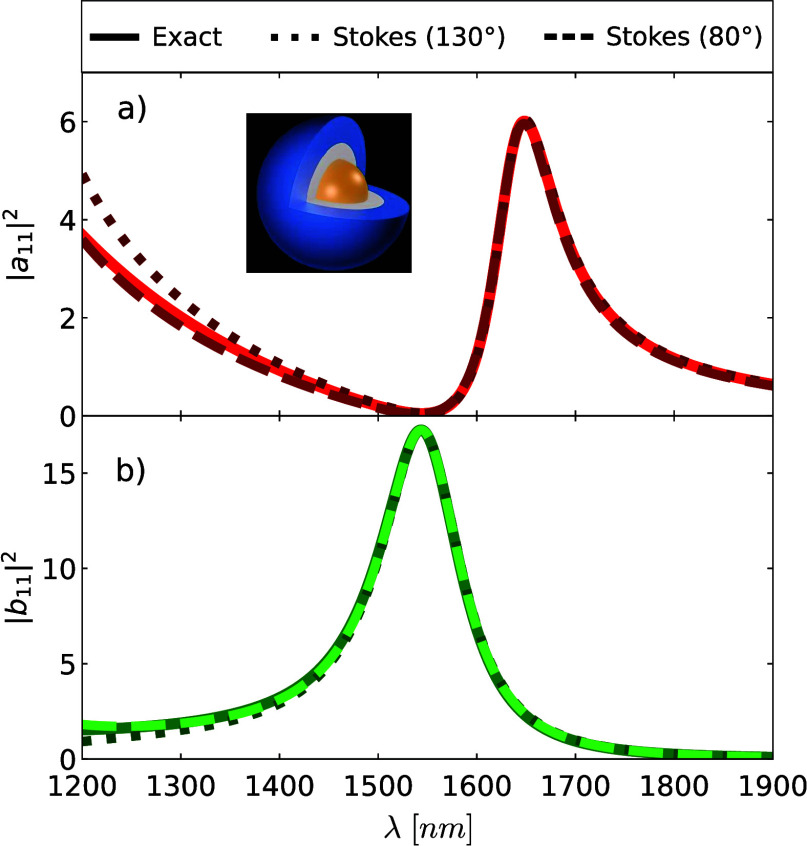
Quadratic combinations of the dipolar electric and magnetic scattering coefficients of an Au core-Ge shell nanoparticle embedded in air with an outer radius *b* = 183 nm and an inner radius *a* = 63 nm, respectively. The incident wavefield is a circularly polarized plane wave. These quadratic forms are calculated from Mie theory (solid) and using the Stokes polarimetry approach summarized in Table 1. Two angles are chosen: θ = 130° (dotted) and θ = 80° (dashed). (a) Electric amplitude |*a*_11_|^2^ depicted in red colors. (b) Magnetic amplitude |*b*_11_|^2^ shown in green colors.

## Methods

The Stokes vector **S** = [*s*_0_, *s*_1_, *s*_2_, *s*_3_] unambiguously describes the polarization state and energy flux of any electromagnetic radiation in the far-field limit.^10^ Importantly, the components of the Stokes vector, typically referred to as the Stokes parameters,^11^ can be measured using a photodiode and conventional wave-plates.^12,13,31,51^ Following Bohren’s and Huffman book,^14^ the Stokes parameters read as
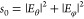
1
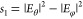
2
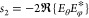
3
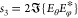
4Here  and  denote the real and imaginary parts, respectively. By inspecting eqs 1-(4), we note that *s*_0_ is the total scattered intensity, *s*_1_ is the degree of linear polarization, *s*_2_ is the degree of linear polarization at 45°, and *s*_3_ denotes the degree of circular polarization.^14^

To determine the four Stokes parameters, we first need to obtain the transversal components of the scattered field evaluated in the radiation (far) zone, namely, *E*_θ_ and *E*_φ_. Hereafter, we follow Jackson’s notation in its third edition to describe the multipolar expansion of the scattered field.^7^ After some algebra (see Supporting Information S1 for the detailed calculation), it can be shown that the scattered field **E**(*k***r**) can be written in the radiation zone (when *kr* → ∞) as
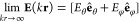
5where
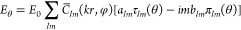
6
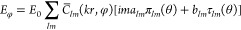
7Here *E*_0_ is the amplitude of the incident wavefield, *k* is the radiation wavenumber, *r* = |**r**| denotes the observation distance to the center of the object, and θ and φ denote the scattering and azimuthal angles, respectively. Moreover, we have defined
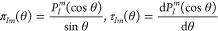
8where  is the Associated Legendre Polynomial^7^ and
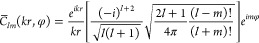
9

At this point we have all the ingredients to calculate the Stokes vector **S**. We recall that the Stokes vector is well-defined if and only if the four Stokes parameters are taken into account. To that end, let us insert eqs 6 and 7 into eqs 1–4, assuming that the object can be fully described by a single multipolar order  and total angular momentum *m*. Notably, several works have tackled such objects in diverse branches of Nanophotonics and Optics. Examples include optically resonant nanoantennas,^15−19^ Kerker conditions,^20−23^ surface-enhanced Raman scattering,^24^ surface-enhanced optical chirality,^13,25^ among many others.^26−29^ Notice that refs (13 and 15−29) are experimental studies widely renowned by the Nanophotonics community. After some algebra, it can be shown that


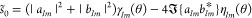
10
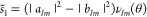
11
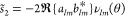
12
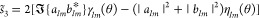
13Here, we have defined  along with
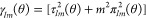
14
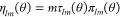
15
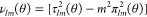
16

Let us briefly discuss the underlying physics behind eqs 10–13. These relations give the dimensionless Stokes vector **S̃** as a function of quadratic combinations of the electric and magnetic scattering coefficients of the multipolar expansion. Note that , , and  do not depend on the optical response of the object and can be straightforwardly determined from eq 8. Now, from eqs 10–13 it is clear that if  and  are known, then the Stokes vector can be calculated. However, in an experiment, one does not have access to  and .

In contrast, and as previously mentioned, the Stokes vector **S** can be measured using a photodiode and conventional wave plates. Therefore, it is convenient to express  and  in terms of **S**. In this regard, it is of utmost importance to note that by simply measuring the Stokes parameters, one cannot distinguish between the electric and magnetic amplitudes of the scattering coefficients. A key step remains to be done to achieve this important distinction. Hereafter, the θ, φ, and *kr* dependence will be assumed. Taking all the previous information into account, we can rewrite eqs 10–13 as
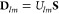
17
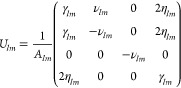
18with , and
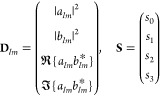
19Equations 17–19 are important results of this work: the quadrivector , which dictates the radiation and coupling between the incident wavefield and the object, can be calculated by measuring the Stokes vector. Importantly, we have not made any assumption on the nature of the spatial distribution of the incident wavefield. Thereby, eqs 17–19 can be applied under a wide variety of illumination conditions: a typical circularly polarized plane wave but also twisted (structured) light such as Gaussian and Laguerre–Gaussian (LG) beams with well-defined angular momentum of light.^30^ Note that the angular momentum must be preserved upon interaction. In ref (30), Zambrana-Puyalto et al. showed that one can excite spherical objects well-described by a single multipolar order using LG beams. In particular, authors proved that LG beams carrying well-defined angular momentum *m* do not excite multipolar orders  in the scattering of the spherical object. Thus, by controlling the optical size and the refractive index contrast of the spherical object, one can ensure that the object is well-described by a single multipolar order. Our method, summarized in eqs 17–19, works for the light-scattering systems in which  for  and *m* > 1. Thus, it can find applications beyond the typical picture of a circularly polarized plane wave impinging on a dipolar spherical object.

Moreover, eqs 17–19 introduce an unprecedented advantage: the capacity to distinguish the electric and magnetic amplitudes of the scattering coefficients. For a clearer understanding, in Table 1 we present the steps to use and implement our Stokes-polarimetry method.

**Table 1:** Receipt to Use the Stokes Vector Measurement to Experimentally Characterize Objects

1. Measure the Stokes vector **S** at an angle θ_1_. Note that this experimental measurement takes into account all multipoles.
2. Calculate the matrix for fixed values of and *m* at θ_1_. An example of the calculation of , for instance, *U*_11_, can be found in the Supporting Information, S2.
3. Use eq 17 to obtain .
4. Repeat steps 1, 2, and 3 for a different scattering angle θ_2_.
5. Compare the values of evaluated at θ_1_ and θ_2_:
5.1. If they resemble each other, the scattering can be fully described by a single multipolar order and *m*, and no additional measurement is needed. In this scenario,is the correct quadrivector: the object has been successfully characterized. In this scenario, we can compute all quadratic combinations of the electromagnetic field in the radiation zone, ranging from far-to-near field.
5.2. If they are different, then the excited object cannot be described by the selected values of and *m*.

## Discussion

To get a deeper insight into the relevance of our findings, we now discuss the features of each of the components that conform .

 and : These scalar terms give full access to the electric and magnetic contribution to the scattering cross-section σ_sca_.^7^ To show this fact, let us derive the scattering cross-section using the standard procedure^7^
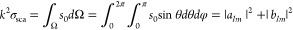
20Equation 20 shows that to determine σ_sca_, *s*_0_ needs to be measured in all directions. This measurement can be achieved using an integrating sphere (see Figure 1), something that is experimentally demanding. Even if we can experimentally measure σ_sca_ using an integrating sphere,^15^ distinguishing between the electric and magnetic amplitudes of the scattering coefficients in σ_sca_ is impossible, both are combined.^15,32−34^ Our analytical findings, summarized in eqs 17−19, provide a solution to this fundamental experimental limitation. We can now determine  and  separately from a measurement of the Stokes vector (see Figure 1b). This advancement allows us to differentiate between electric and magnetic resonances in objects that are well-described by a single multipolar order  and total angular momentum *m*.

At this point, let us provide an illustrative example to show our Stokes polarimetry method in action. In particular, we consider a Au core-Ge shell nanoparticle embedded in air with an inner radius *a* = 63 nm and an outer radius *b* = 183 nm, respectively. We anticipate that this hybrid object fulfills the following feature when exposed to a plane wave: at a certain wavelength, this object behaves as an ideal magnetic dipole.^35^ In other words, at the magnetic dipolar resonance, the electric dipole vanishes. In Figure 2a-b, we show the dipolar electric and magnetic amplitudes of this core–shell nanoparticle, given |*a*_11_|^2^ (see Figure 2a) and |*b*_11_|^2^ (see Figure 2b). These dipolar amplitudes are determined using Mie theory (depicted by solid lines) and using our Stokes polarimetry approach evaluated at θ = 130° (dotted lines) and θ = 80° (dashed lines). Note that other scattering angles could have been selected. As depicted in Figure 2a-b, the calculation of the electric and magnetic amplitudes from the Stokes measurements shows an excellent agreement with the exact calculation in the broadband wavelength interval of 1400 nm < λ < 1900 nm. As we have anticipated, our Stokes polarimetry approach accurately captures the ideal magnetic dipole at λ = 1540 nm. Note that the results obtained from the Stokes polarimetry approach slightly deviate from each other (and from the exact result) at shorter wavelengths, specifically, 1200 nm < λ < 1400 nm. This deviation occurs since, in this wavelength interval, the scattering cannot be fully described by  due to the presence of the magnetic quadrupole. Our Stokes polarimetry approach detects this non-negligible contribution of the magnetic quadrupole, serving as an explicit demonstration of the robustness of our method. Note that numerical methods are not needed to infer the fidelity of our Stokes polarimetry approach. It is self-consistent. For a detailed explanation regarding the fidelity of our Stokes polarimetry approach, read Table 1.

 and . These interference terms, namely,  and , have not been as well-studied as the scattering cross-section in scattering theory. Fortunately, recent developments have shed light on these interference terms within the framework of the Generalized Lorentz Mie theory.^36^ Briefly, the GLMT gives the exact solution of a spherical particle under general illumination conditions.^36^ Considering the GLMT, we can write the interference terms of  as
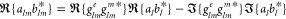
21
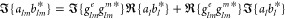
22Note that  and , where  are the electric and magnetic Mie coefficients, respectively, and  are the electric and magnetic coefficients characterizing the incident wavefield, respectively.^37^

**Figure 3:**
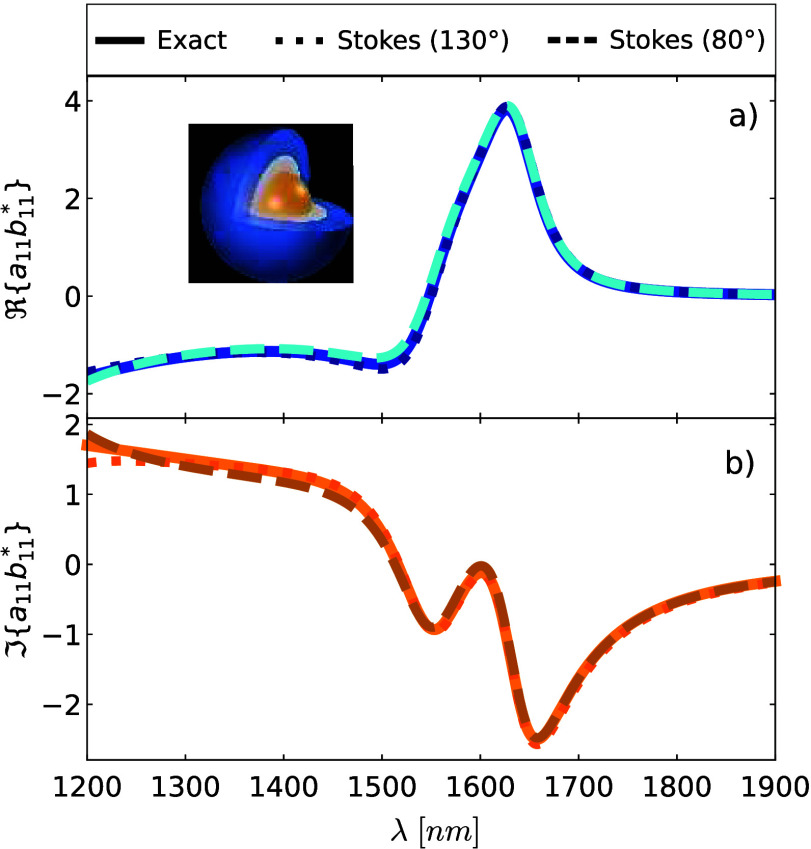
Quadratic combinations of the dipolar electric and magnetic scattering coefficients of an Au core-Ge shell nanoparticle embedded in air with an outer radius *b* = 183 nm and an inner radius *a* = 63 nm, respectively. The incident wavefield is a circularly polarized plane wave. These quadratic forms are calculated from Mie theory (solid) and using the Stokes polarimetry approach summarized in Table 1. Two angles are chosen: θ = 130° (dotted) and θ = 80° (dashed). (a)  plotted in blue colors. (b)  depicted in orange colors.

We now reach notable results: eqs 21 and 22 show that one can retrieve  and  separately upon a Stokes vector measurement. Let us show this by manipulating the helicity of the incident wavefield, with eigenvalues σ = ± 1. First, we note that a wavefield carrying well-defined helicity σ = *m* = +1 satisfies ,^37^ and, hence, yields . In this setting, eqs 21 and 22 are simplified to
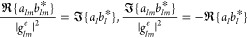
23Equation 23 shows that the interference terms between the electric and magnetic Mie coefficients, namely,  and , can be separately determined from a measurement of the Stokes vector. Indeed, in Figure 3 we show these interference terms, namely,  (see Figure 3a) and  (see Figure 3b), obtained from Mie theory and using our Stokes polarimetry approach evaluated at the previous scattering angles, namely, θ = 130° and 80°. From Figure 3, we can note that there is a remarkable agreement between both calculations in the wavelength interval of 1400 nm < λ < 1900 nm, pointing out that our Stokes polarimetry approach, summarized in eqs 17–19, is suitable to retrieve the interference terms.

To the best of our knowledge, there is currently no alternative method to experimentally measure all these interference terms from a single measurement of the Stokes vector. Thus, eq 23 is an important result of this work. Having noted this important point, these interference terms have recently emerged as key quantities in various branches of Nanophotonics. For instance, the interference term  has shown to be of utmost significance in the preservation of helicity,^38^ Kerker conditions,^20,39−42^ surface-enhanced circular dichroism enhancements,^43^ light transport phenomena,^44^ and optical forces.^45−48^ In stark contrast,  has remained relatively unexplored until recently, primarily appearing in the context of spinless optical mirages^49^ and recoiling optical forces.^48^

Last but not least, let us point out that the determination of  from a far-field measurement of the Stokes vector allows access to the amplitude of the scattered field at all points of the radiation zone. That is, from the far-to-near field. To illustrate this crucial and yet counterintuitive connection, we now write the modulus square of the scattered electric field |**E**|^2^(*k***r**) for objects well-described by fixed values of   and *m*. In this setting, it can be easily shown that (*k***r**) can be written as (see Supporting Information, S1)
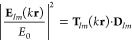
24with .

Equation 24 shows that one can obtain the modulus square of the electric field at any point of the radiation zone if and only if the quadrivector  is determined. Note that the quadrivector  arises from the Hansen multiples and thus, it is a known quantity. As we have accurately captured  from a Stokes vector measurement in the far-field (see Figure 2) we now can compute eq 24 at any point of the radiation zone; particularly, in the near-field limit.

To exemplify this, let us compute the near-field distribution generated by the earlier addressed Au core-Ge shell nanoparticle embedded in air, with an outer radius *b* = 183 nm and an inner radius *a* = 63 nm. Specifically, we calculate the modulus square of the scattered electric field (see eq 24) produced by such hybrid object at a fixed wavelength λ = 1540. In other words, we attain the amplitude of the near-field produced by such an object at the ideal magnetic dipole from a single measurement of the four Stokes parameters.

**Figure 4:**
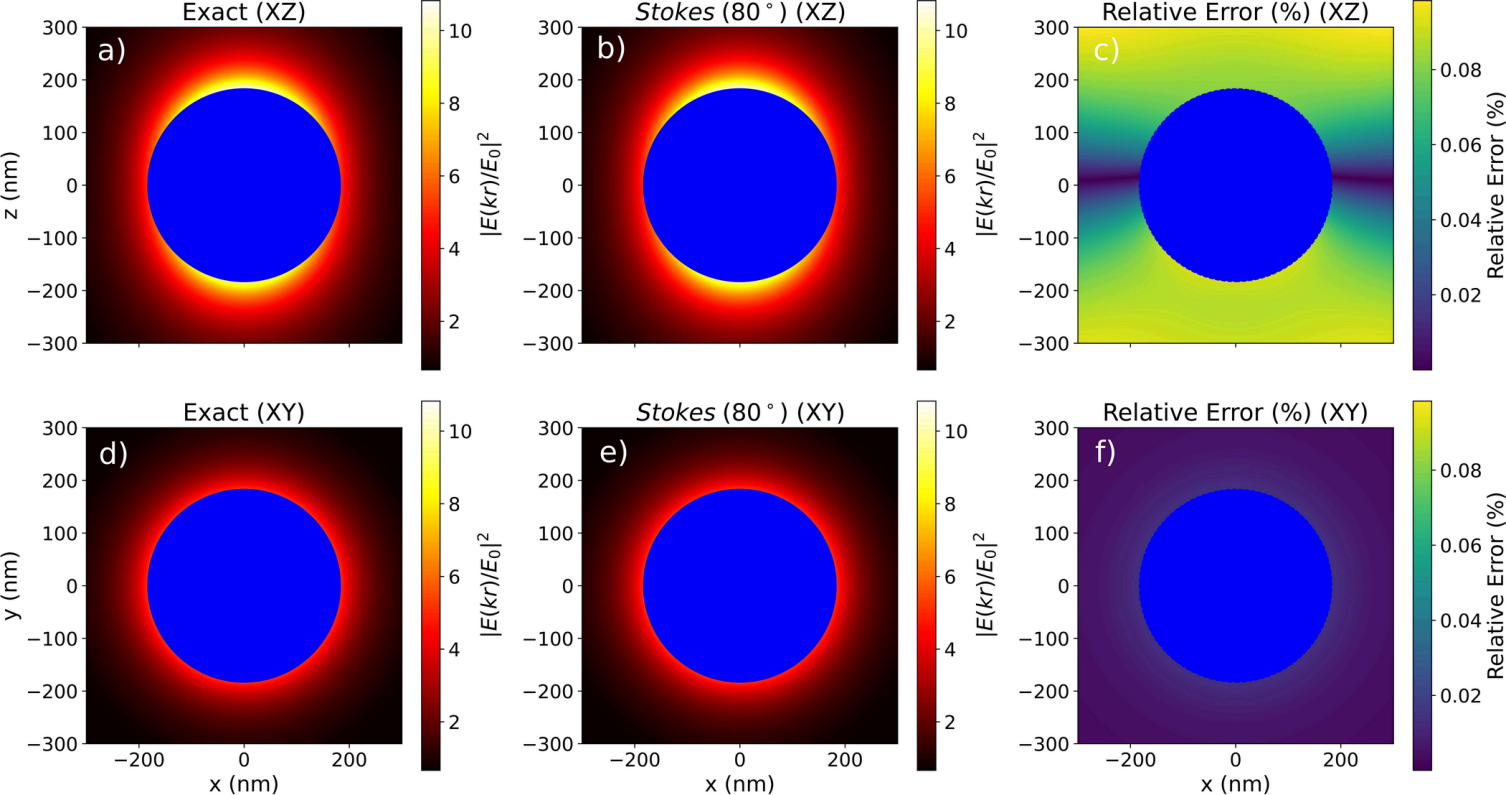
Near-field distributions (in *XZ* and *XY* planes) of an Au core-Ge shell nanoparticle embedded in air, with an outer radius *b* = 183 nm and an inner radius *a* = 63 nm when excited by a circularly polarized plane wave at λ = 1540 nm. The helicity eigenvalue of the plane wave is σ = +1 in all cases. The near-field distribution is calculated from Mie theory (exact) and using the Stokes vector method at θ = 80°. To get deeper insight, the percentage relative error between the exact solution and the Stokes vector method is also shown.

Figure 4 shows the amplitude of the near-field calculated using Mie Theory and using eq 24 at θ = 80°. Note that  has been calculated using our Stokes polarimetry method, summarized in eqs 17–19, and particularized at θ = 80°. The amplitude of the scattered field in the near-field shows an excellent agreement with the exact solution provided by Mie’s theory (exact). This remarkable agreement evidence that the amplitude of the near-field can be captured from a single Stokes vector measurement in the far-field.^50^

To the best of our knowledge, the first connection between local and averaged Stokes parameters was introduced to the physical scene in 2019.^51^ We demonstrated that a local measurement of the intensity (*s*_0_) and degree of circular polarization (*s*_3_) at 90 degrees provides access to the expected (averaged) value of the electromagnetic helicity in the dipolar regime (see Eq. (8) of ref (51)). Later, in 2023, Prof Fujii et al., experimentally corroborated this relationship for a monodisperse solution of silicon nanospheres.^13^ In the same year, we discovered that the relationship between *s*_0_ and *s*_3_ and their averaged counterparts <*s*_0_> and <*s*_3_> applies to any scattering angle of collection.^31^ Moreover, in ref (31), we found that the latter relationship applies for objects well-described by a single  and , expanding its range of applicability for larger spherical objects when excited with structured light.

Having noted this information, we next write our conclusions. We anticipate that all our conclusions (see the forthcoming items) are unattainable using the results of refs (51), (13), and (31).

## Conclusions

In conclusion, we have demonstrated that a measurement of the Stokes vector unlocks key magnitudes at the core of Nanophotonics. These magnitudes are constructed from the quadrivector , captured using our Stokes polarimetry approach. We have shown that the determination of  grants access to the following:

- The separate detection of the amplitudes  and . Remarkably, this distinction is unattainable if measuring the total energy of the scattered field via an integrating sphere. Note that this electric-magnetic distinction is also unreachable if one does not measure the Stokes vector, which accounts the four Stokes parameters.
- The detection of the interference terms between the electric and magnetic Mie coefficients from the same Stokes vector measurement. We have disentangled these interference terms by manipulating the incident helicity of the wavefield.
- The amplitude of the near-field distribution produced by the objects. This can lead researchers to determine reactive quantities from a far-field measurement of the Stokes vector.^48^

## Supporting Information Available

S1: The explicit derivation of the scattered fields in the radiation (far) zone; S2: An example of the  matrix that relates a measurement of the Stokes parameters and the scattering coefficients. In particular, when  (PDF)
