# Supplementary material for: Revealing the Electric and Magnetic Nature of the Scattered Light
Source: ACS Photonics. 2024 Aug 15;11(9):3697–703. doi: 10.1021/acsphotonics.4c00837 (PMC11413847; doi:10.1021/acsphotonics.4c00837)
Supplement: Supplementary file 1 — ph4c00837_si_001.pdf [file ph4c00837_si_001.pdf]

SUPPORTING INFORMATION

Revealing the Electric and Magnetic Nature of the Scattered Light

Jorge Olmos-Trigo*

Departamento de Física, Universidad de La Laguna, Apdo. 456. E-38200, San Cristóbal de La Laguna, Santa Cruz de Tenerife, Spain.

E-mail: jolmostrigo@gmail.com

The Scattered Electromagnetic field in the Far-field

In this Section, we determine the complex amplitudes of the transversal components E_θ and E_φ presented in the main text (see Eqs. (6)-(7)). Let us start by writing the scattered electromagnetic field $\mathbf{E}(k\mathbf{r})$ in terms of electric and magnetic multipoles,[1]

$$\frac{\mathbf{E}(k\mathbf{r})}{E_0} = \sum_{\ell m} \left[a_{\ell m} \boldsymbol{N}_{\ell m}(k\mathbf{r}) + b_{\ell m} \boldsymbol{M}_{\ell m}(k\mathbf{r}) \right]. \tag{1}$$

Here $\boldsymbol{M}_{\ell m}(k\mathbf{r}) = h_\ell^{(1)}(kr)\boldsymbol{X}_{\ell m}(\mathbf{r})$ and $k\boldsymbol{N}_{\ell m}(k\mathbf{r}) = i\nabla \times \boldsymbol{M}_{\ell m}(k\mathbf{r})$ are Hansel multipoles,[1] $\boldsymbol{X}_{\ell m}(\hat{\mathbf{r}}) = \mathbf{L}Y_{\ell m}(\theta,\varphi)/\sqrt{\ell(\ell+1)}$ are vector spherical harmonics, $h_\ell^{(1)}(kr)$ are the spherical Hankel function of the first kind, k is the radiation wavelength, $r = |\mathbf{r}|$ denotes the observation point, θ and φ are the scattering and azimuthal angle, respectively. In this framework, $\mathbf{L} = -i\mathbf{r} \times \nabla$ is the total angular momentum operator and $Y_{\ell m}(\theta,\varphi)$ are spherical harmonics defined as in Ref.[2]

$$Y_{\ell m}(\theta,\varphi) = \sqrt{\frac{2\ell+1}{4\pi}\frac{(\ell-m)!}{(\ell+m)!}} e^{im\varphi} P_\ell^m(\cos\theta), \tag{2}$$

where $P_\ell^m(\cos\theta)$ are the associated Legendre Polynomials.[2] Moreover, a_{lm} and b_{lm} stand for the (dimensionless) electric and magnetic scattering coefficients, respectively, ℓ and m being the multipolar order and total angular momentum of the scattered electromagnetic field introduced in Eq. (1), respectively.

Now, let's calculate Eq. (1) in the far-field limit, namely, when $kr \to \infty$. After algebra, we arrive from Eq. (1) to

$$\frac{\mathbf{E}(k\mathbf{r})}{E_0} = \frac{ie^{ikr}}{kr} \left[(-i)^\ell \left[a_{\ell m} (\hat{\mathbf{r}} \times \boldsymbol{X}_{\ell m}(\hat{\mathbf{r}})) - b_{\ell m} \boldsymbol{X}_{\ell m}(\hat{\mathbf{r}}) \right] \right], \tag{3}$$

where we have made use of the following relations[2]

$$\lim_{kr\to\infty} \boldsymbol{N}_{\ell m}(k\mathbf{r}) = i\frac{e^{ikr}}{kr}(-i)^{\ell}(\hat{\mathbf{r}}\times\boldsymbol{X}_{\ell m}(\hat{\mathbf{r}})), \tag{4}$$

$$\lim_{kr\to\infty} \boldsymbol{M}_{\ell m}(k\mathbf{r}) = \frac{e^{ikr}}{kr}(-i)^{\ell+1}\boldsymbol{X}_{\ell m}(\hat{\mathbf{r}}). \tag{5}$$

At this point, let us express the vector spherical harmonics $\boldsymbol{X}_{\ell m}(\hat{\mathbf{r}})$ in spherical coordinates.[2]

Now, the total angular momentum operator $\mathbf{L}$ reads in spherical coordinates as

$$\mathbf{L} = -i\left[-\hat{\mathbf{e}}_{\theta}\frac{1}{\sin\theta}\frac{\partial}{\partial_{\varphi}} + \hat{\mathbf{e}}_{\varphi}\frac{\partial}{\partial_{\theta}}\right]. \tag{6}$$

Therefore, we can write $\boldsymbol{X}_{\ell m}(\hat{\mathbf{r}})$ in spherical coordinates as

$$\boldsymbol{X}_{\ell m}(\hat{\mathbf{r}}) = \frac{-i}{\sqrt{\ell(\ell+1)}}\left[-\hat{\mathbf{e}}_{\theta}\frac{1}{\sin\theta}\frac{\partial}{\partial_{\varphi}} + \hat{\mathbf{e}}_{\varphi}\frac{\partial}{\partial_{\theta}}\right]Y_{\ell m}(\theta,\varphi). \tag{7}$$

Now, by taking into account Eq. (2), we can write

$$\boldsymbol{X}_{\ell m}(\hat{\mathbf{r}}) = C_{\ell m}(\varphi)\left(-im\pi_{\ell m}(\theta)\hat{\mathbf{e}}_{\theta} + \tau_{\ell m}(\theta)\hat{\mathbf{e}}_{\varphi}\right), \tag{8}$$

$$\hat{\mathbf{r}}\times\boldsymbol{X}_{\ell m}(\hat{\mathbf{r}}) = -C_{\ell m}(\varphi)\left(\tau_{\ell m}(\theta)\hat{\mathbf{e}}_{\theta} + im\pi_{\ell m}(\theta)\hat{\mathbf{e}}_{\varphi}\right), \tag{9}$$

where we have defined

$$\pi_{\ell m}(\theta) = \frac{P_{\ell}^{m}(\cos\theta)}{\sin\theta}, \qquad \tau_{\ell m}(\theta) = \frac{dP_{\ell}^{m}(\cos\theta)}{d\theta}, \tag{10}$$

and

$$C_{\ell m}(\varphi) = \frac{-i}{\sqrt{\ell(\ell+1)}}\sqrt{\frac{2\ell+1}{4\pi}\frac{(\ell-m)!}{(\ell+m)!}}e^{im\varphi}. \tag{11}$$

At this point, let us insert Eqs. (7)-(9) into Eq (3). After some algebraic manipulation, it can be shown that

$$\lim_{kr\to\infty}\mathbf{E}(k\mathbf{r}) = \left[E_{\theta}\hat{\mathbf{e}}_{\theta} + E_{\varphi}\hat{\mathbf{e}}_{\varphi}\right], \tag{12}$$

where

$$E_\theta = E_0 \sum_{\ell m} \bar{C}_{\ell m}(kr,\varphi)\left[a_{\ell m}\tau_{\ell m}(\theta) - imb_{\ell m}\pi_{\ell m}(\theta)\right], \tag{13}$$

$$E_\varphi = E_0 \sum_{\ell m} \bar{C}_{\ell m}(kr,\varphi)\left[ima_{\ell m}\pi_{\ell m}(\theta) + b_{\ell m}\tau_{\ell m}(\theta)\right], \tag{14}$$

where $\bar{C}_{\ell m}(kr,\varphi) = (-i)^{\ell+1}\frac{e^{ikr}}{kr}C_{\ell m}(\varphi)$.

The U_{11} matrix

In this Section, we calculate, step by step, the U_{11} matrix. By inspecting Eq. (18) we need the Associated Legendre Polynomials $P_{\ell m}(\cos\theta)$. Hereafter, we follow the notation of Jackson's book in its third edition.[2] When setting $\ell = m = 1$, we get $P_{11}(\cos\theta) = -\sin\theta$. Then, we insert this into Eq. (8) of the main text to obtain, $\tau_{11} = -1$ and $\pi_{11} = -\cos\theta$. Then, we insert these values in Eqs. (14-16) yielding

$$\gamma_{11} = 1 + \cos^2\theta, \qquad \eta_{11} = \cos\theta, \qquad \nu_{11} = -\sin^2\theta. \tag{15}$$

At this point, only a step remains to be done to calculate U_{11} matrix: we need to compute $A_{11} = 2\nu_{11}^2|E_0|^2|C_{11}|^2$. To achieve this goal, we must compute $|C_{11}|^2$. This expression can be found in Eq. (9). Now, by setting $\ell = m = 1$, we get

$$|C_{11}|^2 = \frac{3}{16\pi}\frac{1}{(kr)^2} \tag{16}$$

At this point, we have all the ingredients to calculate the U_{11} matrix. Taking into account Eqs. (15)-(16), we arrive to

$$U_{11} = \frac{1}{A_{11}} \begin{pmatrix} 1+\cos^2\theta & -\sin^2\theta & 0 & 2\cos\theta \\ 1+\cos^2\theta & \sin^2\theta & 0 & 2\cos\theta \\ 0 & 0 & \sin^2\theta & 0 \\ 2\cos\theta & 0 & 0 & 1+\cos^2\theta \end{pmatrix}, \tag{17}$$

where $A_{11} = \dfrac{3|E_0|^2 \sin^4\theta}{8\pi(kr)^2}$.

References

(1) Olmos-Trigo, J.; Lasa-Alonso, J.; Gómez-Viloria, I.; Molina-Terriza, G.; García-Etxarri, A. Capturing near-field circular dichroism enhancements from far-field measurements. *Physical Review Research* **2024**, *6*, 013151.

(2) Jackson, J. D. *Classical Electrodynamics*; John Wiley & Sons, New York, 1999.
